# Supplementary material for: Niche-derived soluble DLK1 promotes glioma growth
Source: Neoplasia. 2020 Oct 23;22(12):689–701. doi: 10.1016/j.neo.2020.10.005 (PMC7587507; doi:10.1016/j.neo.2020.10.005)

**Supplementary**

**Supplementary Figure 1**

A-B: Representative images of immunofluorescent stainings showing tumor-associated astrocytes and DLK1 localization in perinecrotic (N) and perivascular (V) areas (A and B respectively) of shp53-induced murine gliomas.

C: Representative images of immunofluorescent stainings showing tumor-associated astrocytes and N-terminal, secreted, DLK1 localization in perinecrotic areas (N) of shp53-induced murine gliomas.

Scalebars represent 25µm.

**Supplementary Figure 2**

Validation of anti N-terminal DLK1 antibody showing specific signal in perinecrotic areas of shp53-induced murine gliomas and absent or very low signal in bulk tumor areas and healthy brain. No signal was detected with secondary antibody control.


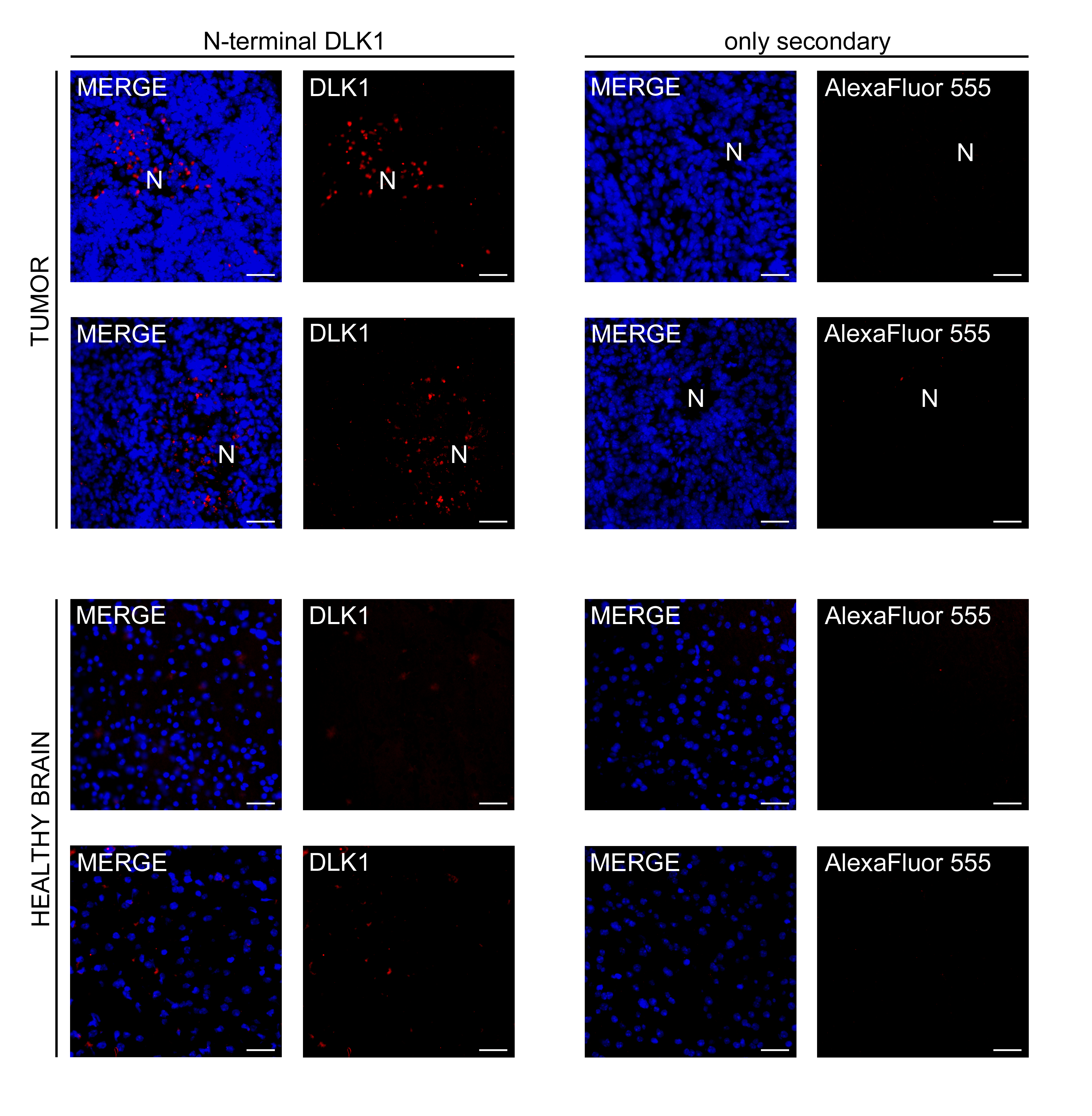


**Supplementary Figure 3**

Representative images of primary sphere forming assays in U3082MG seeded at different concentrations showing the formation of single, well defined clonal spheres in clonal concentrations (first and second column) and the presence of less defined spheres together with cell aggregates at higher concentrations (third and fourth column).


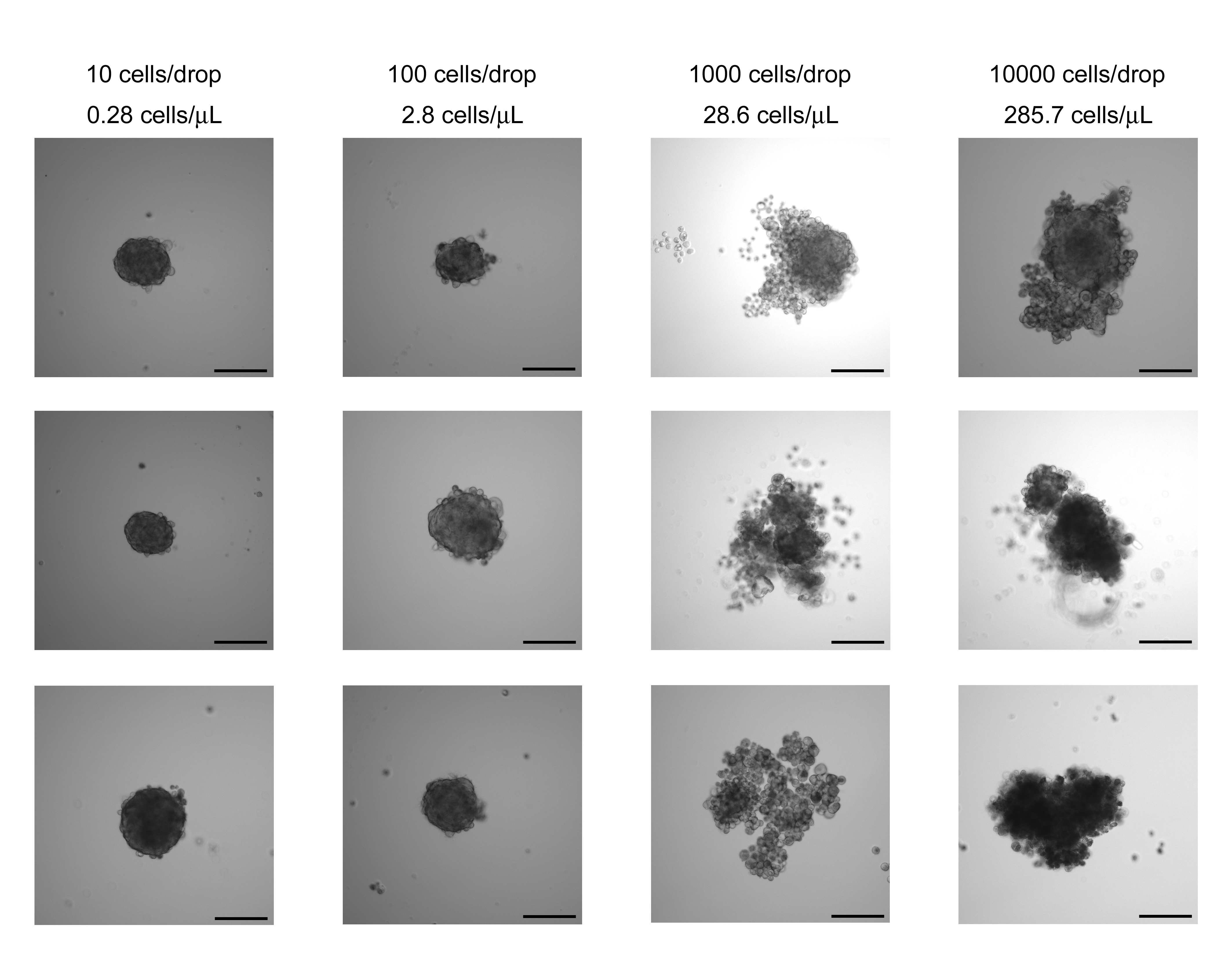


**Supplementary Figure 4**

Representative images and densitometric analysis of western blots showing HIF-1a and HIF-2a in cells in cells untreated or treated with 50 ng/ml recombinant DLK1, 10 µM PT2385 and grown in 21% or 1% O_2_ for 72 hours. Results are expressed as fold changes of respective normoxic controls.

Statistical analysis: n=3. All data are expressed as mean±SEM. Statistical significance was determined by one-way ANOVA followed by Bonferroni post hoc test. In the whole figure significance is represented as ** p<0.01 and *** p<0.001 as indicated by straight lines


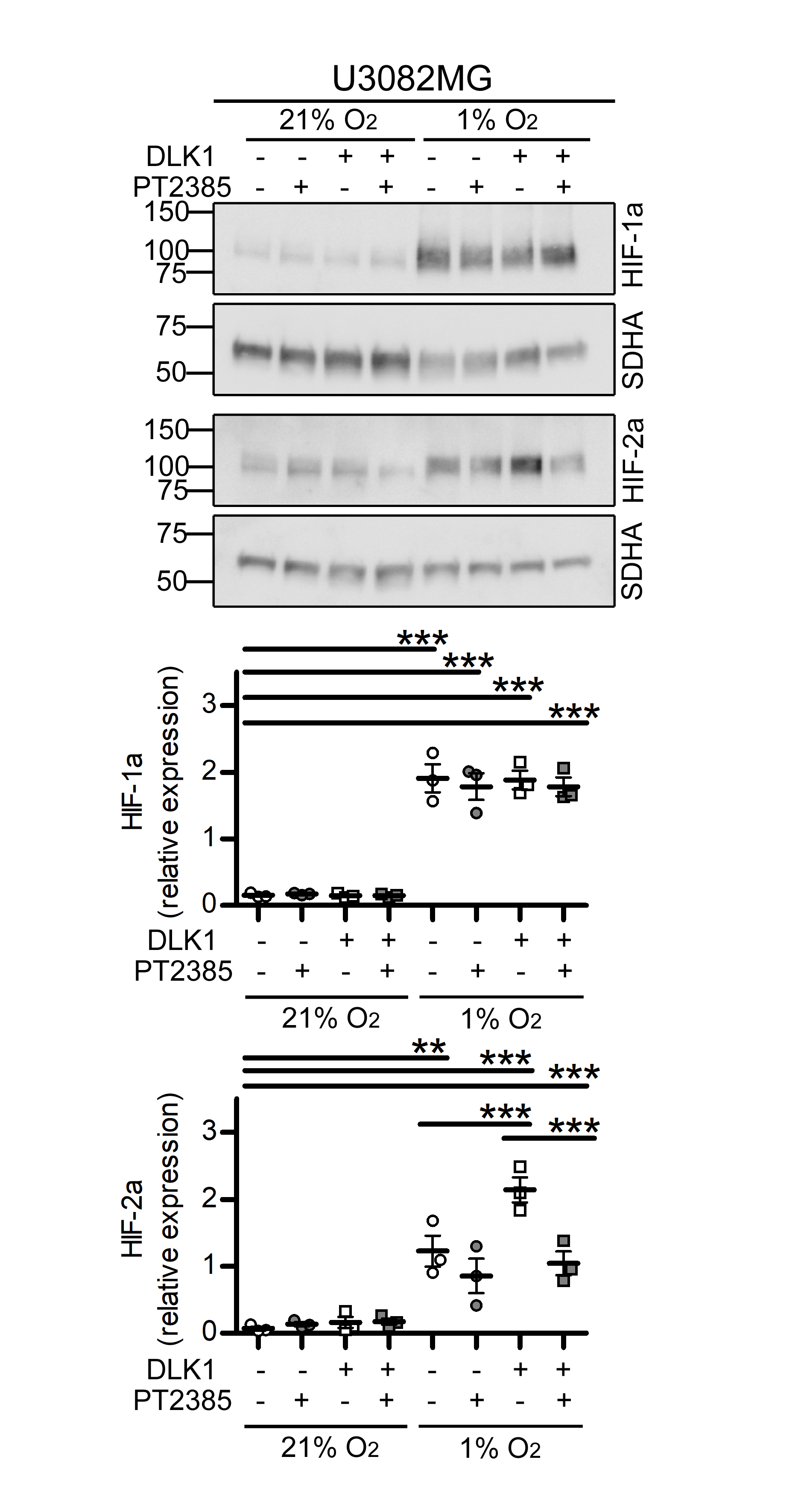


**Supplementary Figure 5**

Kaplan-Meier survival plot of DLK1 high vs. low tumors in the indicated datasets for GBM only (A) or all glioma (B).


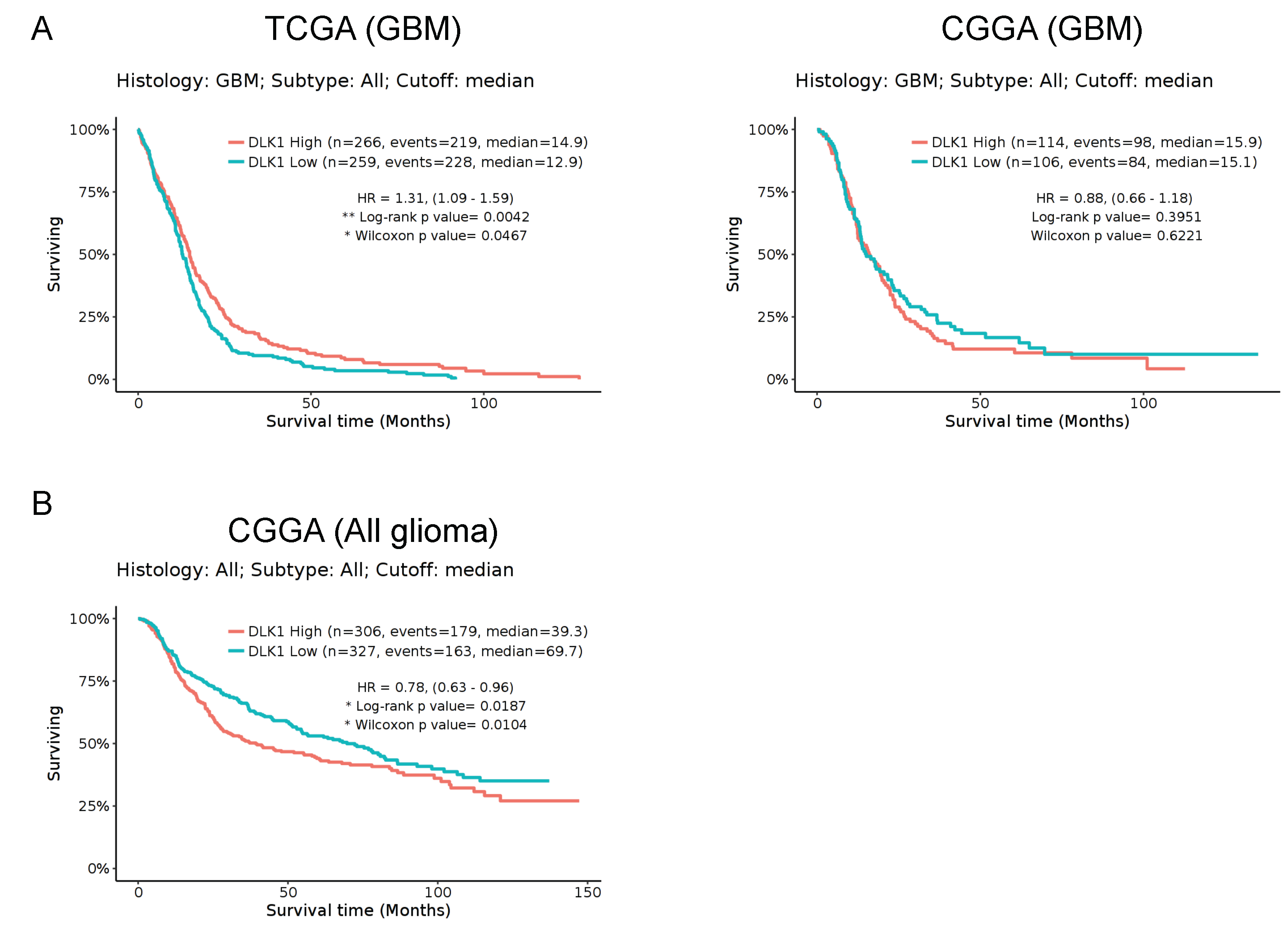

Supplement: Supplementary file 1 [file mmc1.docx]
